# Reactions of Pd(II) and Pt(II) Complexes With Tetraethylthiouram Disulfide

**DOI:** 10.1155/MBD.1997.317

**Published:** 1997

**Authors:** G. Cervantes, V. Moreno, E. Molins, C. Miravitlles

**Affiliations:** 1 Departament de Química Inorgànica Universitat de Barcelona Avgda Diagonal, 647 Barcelona 08028 Spain; 2 Institut de Ciència de Materials de Barcelona CSIC Bellaterra Cerdanyola 08193 Spain

## Abstract

The reactions of tetraethylthiouram disulfide (DTS), an inhibitor of the nephrotoxicity of Pt(II) drugs, an efficient agent in the treatment of chronic alcoholism, in the treatment of HIV infections, AIDS and heavy metal toxicity, and a fungicide and herbicide, with K_2_[PtCl_4_], in ratio 1:1 and 1:2, gave the compounds [PtCl_2_DTS] and [Pt(S_2_CNEt_2_)_2_] respectively. The reaction of the complexes K_2_[PdCl_4_], Pd(AcO)_2_ and [PdCl_2_(PhCN)_2_], where PhCN = Benzonitrile, with tetraethylthiouram disulfide in ratio 1:1 or 1:2, yielded orange crystals identified as [Pd(S_2_CNEt_2_)_2_]. The crystals were suitable for study by X-ray diffraction. The -S-S- bridge in the tetraethylthiouram disulfude molecule was broken and the two molecules of the thiocarbamate derivative were bound to the Pd(II) by the equivalents sulfur atoms. All the compounds were characterized by IR, ^1^H and ^13^C NMR spectroscopies.


### REACTIONS OF Pd(II) AND Pt(II) COMPLEXES WITH TETRAETHYLTHIOURAM DISULFIDE

G. Cervantes[1], V. Moreno[2*], E. Molins[2] and C. Miravitlles[2]

[1] Departament de Química Inorgànica Universitat de Barcelona.
Avgda Diagonal, 647, 08028-Barcelona. Spain
[2] Institut de Ciència de Materials de Barcelona, CSIC, Bellaterra, 08193- Cerdanyola, Spain

**Abstract**

The reactions of tetraethylthiouram disulfide (DTS), an inhibitor of the nephrotoxicity of Pt(II) drugs, an efficient agent in the treatment of chronic alcoholism, in the treatment of HIV infections, AIDS and heavy metal toxicity, and a fungicide and herbicide, with $K_2[PtCl_4]$, in ratio 1:1 and 1:2, gave the compounds $[PtCl_2DTS]$ and $[Pt(S_2CNEt_2)_2]$ respectively. The reaction of the complexes $K_2[PdCl_4]$, $Pd(AcO)_2$ and $[PdCl_2(PhCN)_2]$, where PhCN = Benzonitrile, with tetraethylthiouram disulfide in ratio 1:1 or 1:2, yielded orange crystals identified as $[Pd(S_2CNEt_2)_2]$. The crystals were suitable for study by X-ray diffraction. The -S-S- bridge in the tetraethylthiouram disulfude molecule was broken and the two molecules of the thiocarbamate derivative were bound to the Pd(II) by the equivalents sulfur atoms. All the compounds were characterized by IR, $^1H$ and $^{13}C$ NMR spectroscopies.

**Introduction**

*Cisplatin* and other Pt(II) complexes are nephrotoxic. A number of nucleophilic agents, mainly sulfur compounds, inhibit these toxic effects[1-3]. The interaction of *cisplatin* with several of these compounds such as methionine, penicillamine and glutathione, has been studied in recent years[4-13].

To elucidate the mechanisms involved in the inhibition of toxicity, we have studied the behavior of Pt(II) complexes containing ligands with -S-S- bonds such as tetraethylthiouram disulfide [DTS,bis(diethylthiocarbamoyl) disulfide, 1,1'-dithiobis(N,N'-diethylthioformamide), dithiosulfiram, Antabuse®, Noxal®, Abstensil®, BAN] **(1)**. This compound is used in the treatment of the chronic alcoholism, as a fungicide and herbicide, and also to inhibit the secondary effects of the *cisplatin*, in the treatment of HIV infections, AIDS and heavy metal toxicity[14-15]. The study was also extended to the interaction of the tetraethylthiouram disulfide molecule with Pd(II) complexes.

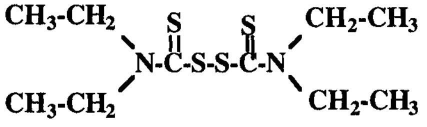

(1)

**Scheme 1. Tetraethyldithiouram disulfide (DTS)**

On the other hand, the diethyldithiocarbamate molecule (DEDTC, Imuthiol®) **(2)**, related with tetraethylthiouram disulfide is present in rubbers and plastics[16] and it has also been used as inhibitor of *cisplatin* toxicity without inhibition of the antitumor activity[17,18]. This may be due to its high affinity for Pt, which causes the breaking of Pt-protein adducts[19] without capture of the Pt bound to DNA[20]. Pt(II)-diethyldithiocarbamate complexes have been found in plasma of patients treated with this inhibitor[21].

Both subtances, tetraethylthiouram disulfide (DTS) and its derivative diethyldithiocarbamate (DEDTC) have similar applications as fungicides, pesticides, antioxidants, lubricants, flotation agents, and vulcanization accelerators[22] and they are active against some typus of leukemia, probably due to their immunomodulation properties[23]. The mutual interconversion is easily produced, especially in the biological medium[20] and inside lubricants DTS is also converted into DEDTC[16].

The crystal structure of tetraethylthiouram disulfide was studied previously[24]. The most important feature of this molecule is that, although there is no $C_2$ symmetry, the two halves are chemically equivalent. Wang *et al.* had shown interest in deformation density studies[25] due to the short distances and forced angles found at room temperature. However, these studies showed a low electronic density in the zone between the two bound S atoms. This feature facilitates the breaking of the molecule at this point, and two dithiocarbamate ions are produced. Studies on the thermal dissociation of DTS showed that the radical free process is reversible and that the solvent has no influence on it[26]. At 120ºC DTS dissociates to DEDTC. The process can be reversed by the action of cytochrom c or hydrogen peroxide, among others[20].

Due to the proclivity of DTS to dissociate to DEDTC, only a few compounds of DTS have been described. Brinkhoff et al.[27] synthesized derivatives of Hg, Cuadrado *et al.* [28] compounds of Ti and V and Contreras *et al.*[29] complexes of Cr(III). Pd(II) and Pt(II) derivatives of tetramethylthiuram disulfide were obtained[30] but not spectroscopically characterized. Only one study on crystal structure has been reported for a methyl derivative of DTS, $[HgI_2(Me_4DTS)]$[31]. In this compound, the $Me_4DTS$ is coordinated to the metal in bidentate mode by the two sulfur atoms from the C=S groups.

We have studied the reactions of tetraethylthiouram disulfide with $K_2[PdCl_4]$, $Pd(AcO)_2$ , $[PdCl_2(PhCN)_2]$, where PhCN = Benzonitrile, and $K_2[PtCl_4]$. In some cases, we have observed the breaking of the S-S bond of the tetraethylthiouram disulfide to give two molecules of the corresponding dithiocarbamate, which binds to the metal ion giving a very stable complex as a product, with similar structural characteristics to the Ni(II) diethyldithiocarbamates studied by Bonamico *et al.*[32-34], Pd(II) by Gessner *et al.*[15], the Pt(II) by Amanov *et al.*[35] and the Mo(V) diethyldithiocarbamate compound characterized by Kocaba *et al.*[36]. No $[M(DEDTC)_2]$ (M= Pd, Pt) compound has yet been described from reaction with DTS.

### Experimental

### *Materials and Methods*

The complexes were prepared using $K_2[PdCl_4]$, $Pd(AcO)_2$ and $K_2[PtCl_4]$ products from Johnson Matthey and tetraethylthiouram disulfide from Sigma. The $[PdCl_2(PhCN)_2]$ complex was prepared by reaction of $PdCl_2$ (Johnson Matthey) with benzonitrile (Fluka) at reflux during five hours. The product was recrystallized of chloroform or THF.

Elemental analyses were carried out on a Carlo Erba 1500 microanalyzer at the Serveis Científico-Tècnics at the University of Barcelona. The infrared spectra were recorded in solid state (KBr pellets) on a FT-IR Nicolet 5DZ spectrometer in the 4000-400 $cm^{-1}$ range and on a FT-IR Bomem DA-3 spectrometer in the 400-150 $cm^{-1}$ range. $^1H\{^{13}C\}$ and $^{13}C\{^1H\}$ NMR spectra were obtained on a Varian Gemini 300 spectrometer using $CDCl_3$ as solvent. Chemical shifts were measured relative to TMS.

Suitable crystals for X-ray diffraction experiments were mounted on an Enraf-Nonius CAD4 four-circle diffractometer. Unit cell parameters were determined from 25 reflections and refined by the least-squares method. Intensity data were collected using graphite monochromated MoKα radiation. Lorentz and polarization corrections were applied but not corrections for absorption due to the small volume of the crystal selected. The structure was solved locating the Pd atom by direct methods using the MULTAN 11/84 program[37]. The positions of the remaining non-hydrogen atoms were determined by weighted Fourier synthesis. Refinement was carried out using the SHELX-76 program[38]. Hydrogen atoms were located by difference Fourier synthesis and introduced in the refinement with a global isotropic temperature factor after the convergence of the anisotropic thermal parameters for non-H atoms. Methyl groups were allowed to rotate axially in the last stages of refinement.

### *Syntheses of the Complexes*

*(a)* $[Pd(S_2CNEt_2)_2]$ (**3**). 1 mmol of $K_2[PdCl_4]$ and 2 mmol of tetraethylthiouram disulfide (DTS) were dissolved in 20 mL of 50% mixture ethanol/water. The solution was stirred for 1 h at 40 ºC and then a yellow precipitated appeared. The solid was filtered, washed in ethanol and dried overnight under silica gel. Found: C, 29.53; N, 7.06; S, 32.51. $PdC_{10}H_{20}N_2S_4$ requires: C, 29.81; N, 6.95; S, 31.83. When the compound (**3**) was recrystallized in THF or in chloroform, bright orange crystals were formed. These crystals were suitable for study by X-ray. Found: C, 29.80; N, 6.78; S, 32.13. $PdC_{10}H_{20}N_2S_4$ requires: C, 29.81; N, 6.95; S, 31.83. The reactions of the complexes $Pd(AcO)_2$, and $[PdCl_2(PhCN)_2]$ with DTS in 1:2 ratio in chloroform or THF, also yielded similar bright orange crystals identified as $[Pd(S_2CNEt_2)_2]$. The reaction in the 1:1 ratio gave a brown solid, possibly a Pd(II) DTS derivate, which in THF or chloroform yielded the bright orange crystals of $[Pd(S_2CNEt_2)_2]$ and a dark residue. Other attempts to obtain a Pd(II)-DTS, by changing the solvent, or in absence of oxygen failed and $[Pd(S_2CNEt_2)_2]$ was always identified as final product.

*(b)* $[PtCl_2(DTS)]_2.10H_2O$ (**4**). 1 mmol of $K_2[PtCl_4]$ and 1 mmol of tetraethylthiouram disulfide were dissolved in 20 mL of 50% mixture ethanol/water. The solution was stirred for 1 h at 40 ºC and a dark brown precipitated was formed. The solid was filtered, washed in ethanol and dried overnight under silica gel. Found: C, 18.44; N, 4.16; S, 20.63; Cl, 10.05. $Pt_2C_{20}H_{60}N_4S_8Cl_4O_{10}$ requires: C,18.46; N, 4.31; S, 19.71; Cl, 10.90. All efforts to obtain crystals suitable for study by X-ray were unsuccessful. Only macled needles could be isolated.

*(c) [Pt(S$_2$CNEt$_2$)$_2$]* (**5**) 1 mmol of $K_2[PdCl_4]$ and 2 mmol of finely powdered tetraethylthiouram disulfide were mixed and 20 mL of ethanol was added. The solution was stirred for 1 h at 40 ºC and a pale pink precipitate formed, which evolved to dark brown. After 36 h stirring at room temperature the solid was filtered but it could not be identified. Brown needles suitable for X-ray diffraction were obtained from the solution. Found: C, 24.87; H, 4.12; N, 5.95; S, 25.94. $PtC_{10}H_{20}N_2S_4$ requires: C, 24.43; H, 4.07; N, 5.70; S,. 26.02.

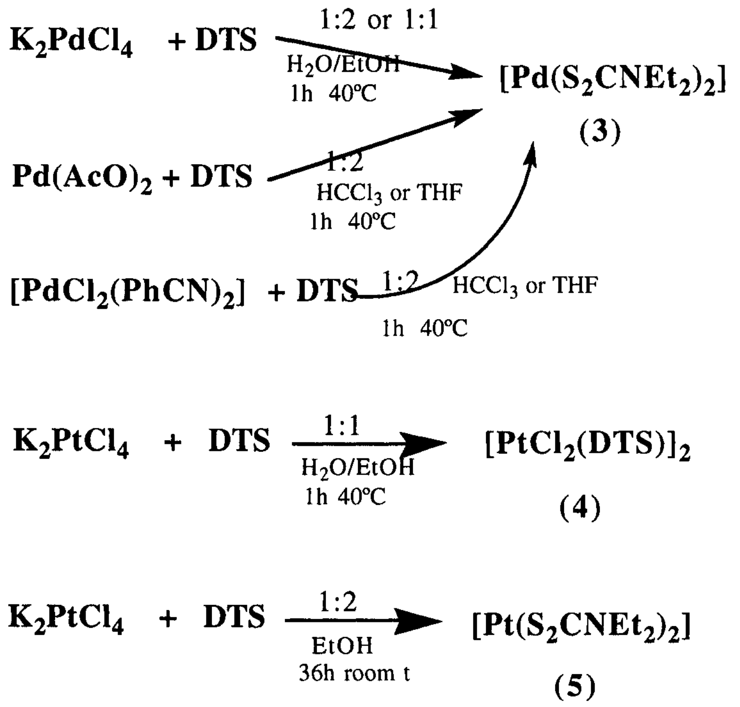

**Scheme 2. Reactions of tetraethylthiouram disulfide with Pt(II) and Pd(II) complexes**

### Results and discussion

### *FTIR study*

The main IR frequencies of the tetraethylthiouram disulfide, the diethyldithiocarbamate and their Pd(II) and Pt(II) complexes obtained are reported in Table I.

The spectra of tetraethylthiouram disulfide and diethyldithiocarbamate molecules are very similar. The main difference is the presence of a band at 434 $cm^{-1}$ in the spectrum of the DTS. This band is assigned to the stretching mode ν(S-S)[39,40]. In the spectrum of (**4**) this band appears but it is absent in the spectra of (**3**) and (**5**), confirming the breaking of the S-S bond. The band assigned to $\nu_{CN}$ and $\nu_s$(CNC) coupled with the inner modes from alkyl groups[41], also called thioureid band[42,43] appears at 1497 $cm^{-1}$ in DTS and at lower frequency in free DEDTC. This band moves to higher frequencies (30-40 $cm^{-1}$ for DTS and 40-50 $cm^{-1}$ for DEDTC) in the new complexes. This is due to the ability of the amines to transfer density of charge towards the S atoms through the π system, thus reforcing the C-N bond[44]. In the case of dithiocarbamates, the resonant form IV (Scheme 3) explains the shift of the band towards higher frequencies[42].

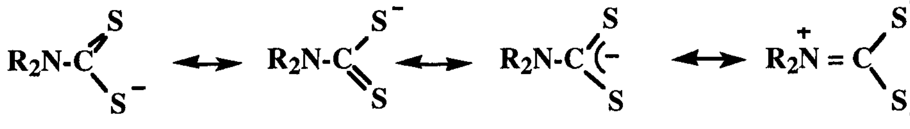

(I) (II) (III) (IV)

**Scheme 3. Resonants forms for dialkyldithiocarbamate anions**

**Table 1.** IR frequencies ($cm^{-1}$) for Pd(II) and Pt(II) derivatives of DTS and DTDTC

| Assignment | DTS | Na-DEDTC | Pd-DEDTC | Pt-DTS | Pt-DEDTC |
|---|---|---|---|---|---|
| ν(C-N) and $\nu_s$(C-N-C) | 1497vs | 1476s | 1520vs | 1533vs,br | 1526vs |
| ν(C-N) | 1296m | 1297m | 1299m | - | 1300w |
| $\nu(C\text{-}S)_{asym}$ | 1001m | 984s | 988m | 987m,br | 987m |
| $\nu(C\text{-}S)_{sym}$ | 555m | 566m | 570m | 574w<br>554w | 568m |
| ν(S-S) | 434w | - | - | 438w | - |
| ν(M-S) | - | - | 356m | 330m,b | 340m |
| ν(M-Cl) | - | - | - | 324m<br>306m | |

The resonant forms which contribute to the electronic structure for the DTS complexes are shown in Scheme 4. The lesser contribution is that of the form IV because the band ν(CN) is absent in the complexes of DTS.

The band at 1296 $cm^{-1}$ assigned to ν(CN) appears in the spectra of **(3)** and **(5)** but not in the spectrum of **(4)**, confirming the formation of DEDTC complexes in the former and of DTS complex in the latter[39]. The stretching frequency $\nu(C\text{-}S)_{asym}$[41,44] appears at 1000 $cm^{-1}$ for DTS and at 987 $cm^{-1}$ for DEDTC. In the Pd(II) and Pt(II) complexes of DEDTC only one band appears in this zone, as corresponds to bidentate dithiocarbamates[42]. In the spectrum of **(4)** only one band is observed which also indicates bidentate coordination for DTS[28,45]. This band shifts to lower frequencies as a consequence of the lengthening of the end C-S bond when it is coordinated to the metal ion. The vibration $\nu(C\text{-}S)_{sym}$ appears as a single band at 555 $cm^{-1}$ in the DTS spectrum and at 566 $cm^{-1}$ in the DEDTC spectrum. The band appears split in the spectrum of **(4)**, but not in the spectrum of **(3)** or **(5)**. In all cases this band is shifted towards higher frequencies in the complexes. The new bands that appear at low frequencies are assigned to ν(M-S) and ν(M-Cl), (Table 1).

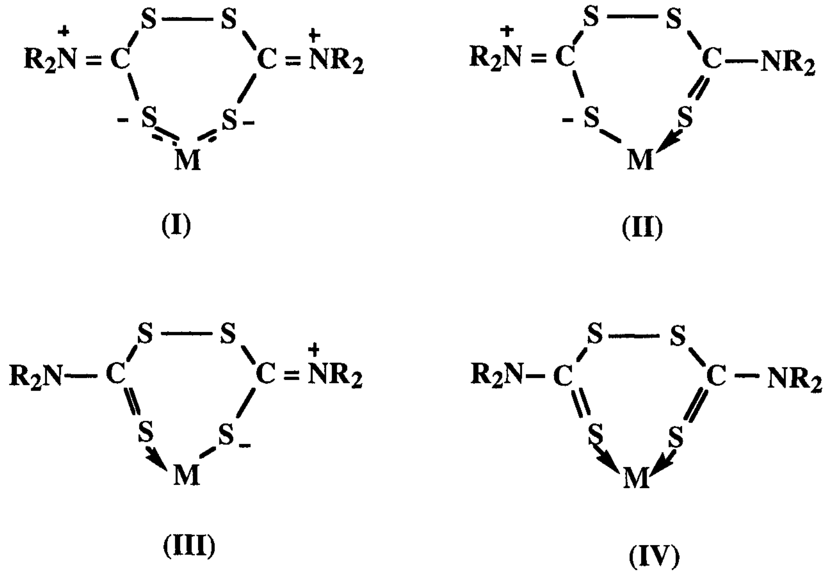

**Scheme 4. Resonants forms for dialkyldithiouram disulfide complex**

### *X-Ray Study*

Crystal parameters and a summary of the data collection and refinement process corresponding to compound (3) are given in Table 2. Although the cell parameters suggest a tetragonal cell, the analysis of the equivalence between symmetry related reflections shows that the cell is monoclinic. A perspective view of the molecule, including the atom labeling, is shown in Figure1. Fractional atomic coordinates with the equivalent temperature factors are listed in Table 3. Table 4 contains the corresponding bond distances and angles with their esd. Anisotropic thermal parameters and the listing of observed and calculated structural factors may be obtained on request from the authors. Crystal packing is depicted in Figure 2. The Pd atoms are situated in the crystallographic center of the cell.

The palladium atom is in planar coordination, the Pd-S distances being equivalent. The angles do not correspond exactly to a square distribution but a rhombus. For example, for the $Pd_1$ atom one, the angles $S_{12}$-$Pd_1$-$S_{11}$ and $S'_{12}$-$Pd_1$-$S'_{11}$ are equal to 75.6º and the $S_{12}$-$Pd_1$-$S'_{12}$ and $S_{11}$-$Pd_1$-$S'_{11}$ are equal to 104.4º. There are two crystallographically non-equivalent molecules (Figure 1, a and b) which have the ethyl groups in different orientation but the distances and angles of both molecules have the same values. The $C_{21}$-$N_{22}$ and $C_{11}$-$N_{12}$ distances are shorter than expected indicating bond order greater than one. This fact is consistent with the IR data commented above. The dithiocarbamate C-S distances are also equivalent in all cases, indicating the delocalization of the anion charge produced when the initial molecule of dithiosulfiram breaks in the presence of the Pd(II) ion. The bond order is smaller in this case than in the whole ligand.

The crystal data of this Pd(II) complex was compared with that from the crystal structure of the tetraethylthiouram disulfide[24,25] and with Ni(II), Cu(II), Zn(II), and Mo(V) diethyldithiocarbamates complexes reported in the literature[32-34,36]. The C-S distances found for the Pd(II) compound range from 1.735 Å to 1.715 Å, which is very similar to the values for the Ni(II), Cu(II) and Zn(II) diethyldithocarbamate complexes, which are between 1.700 Å and 1.725 Å. However, the C-N distance in the Pd(II) complex, 1.294 Å, is shorter than that corresponding to the Ni(II), Cu(II) and Zn(II) derivatives, which are between 1.35 Å and 1.33 Å, and shorter than the C-N distances in each perpendicular half of the tetraethylthiouram disulfide molecule, 1.33Å and 1.36 Å. Thus, the bond order for C-N is higher in the Pd(II) compound than in tetraethylthiouram disulfide and the other complexes. The other lengths are similar and as expected in this type of compound.

The data corresponding to $[Pd(S_2CNEt_2)_2]$ **(3)**, can be also compared with $[Pt(S_2CNEt_2)_2]$, **(5)**. In the reaction of $K_2[PdCl_4]$ tetraethylthiouram disulfide in ethanol (1:2), brown needles suitable for X-ray study were isolated and identified as the compound of platinum and diethyldithiocarbamate described by Baker[46]. The resolution of the structure confirmed the breaking of the S-S atom in DTS, which gave compound **(5)**. The C-N length in $[Pt(S_2CNEt_2)_2]$ is 1.32 Å, while the C-N length in $[Pd(S_2CNEt_2)_2]$ is 1.29 Å; therefore, the bond order is lower than that of the Pd compound, but slightly higher than that corresponding to the DTS molecule.

**Table 2**. Crystal data and Summary of Data collection and refinement for $[Pd(S_2CNEt_2)_2]$

| | |
|---|---|
| Crystal system | Monoclinic |
| Space group | P2/n |
| a(Å) | 16.430 (2) |
| b(Å) | 6.237 (1) |
| c(Å) | 16.430 (2) |
| β(º) | 90.00 (1) |
| V(Å$^3$) | 1683.5 |
| Z | 4 |
| $D_c$(gcm$^{-3}$) | 1.59 |
| Crystal size (mm) | 0.45 x 0.37 x 0.25 |
| F(000) | 884 |
| μ(cm$^{-1}$) | 15.3 |
| Radiation | $MoK_\alpha$(λ=0.71073Å) |
| Scan method | w-2θ |
| Data collection range(2θ) | 2-60.8° |
| Range of hkl | -22<h<22, 0<k<8, 0<l<23 |
| N. of measured refl. | 5160 |
| N. of unique refl. | 4804 |
| N. of obs.refl.(I≥2σ(I)) | 4108 |
| N. of variables | 170 |
| R | 0.031 |
| $R_w$ | 0.048 |
| Weighting scheme k, $w=1/(\sigma^2(Fo)+ kF^2$ | 0.018084 |

Study of the electronic densities at low temperature in tetramethyl and tetraethylthiouram disulfides carried out by Wang *et al*[25], explains the breaking of the S-S bond to give two negative halves. The shorter C-N bond gives greater density accumulation (0.5 e Å$^{-3}$) at the midpoint of the bond than is observed for the longer bonds (0.3 e Å$^{-3}$). The C=S double bond gives 0.4 e Å$^{-3}$ whereas the C-S single bond is 0.2 e Å$^{-3}$. There is little density accumulation along the S-S bond.

Lone-pair electron density is apparent around all the S atoms. The degree of the density accumulation at the midpoint of bonded atoms follows the order: shorter C-N > C-C > longer C-N, C=S > C-S > S-S. The soft metal ion Pd(II) can easily coordinates to the S atoms to produce the stable neutral dithiocarbamate complex.

The non equivalence of the protons of $-CH_2-$ and $-CH_3$ can be observed in the spectrum of DTS. In the case of $CH_2$, instead of the expected quadruplet, a multiple appears. Likewise, two triplets can be observed for the $CH_3$ groups. This means that the two ethyl groups from $SC(S)N(Et)_2$ are not equivalent as a consequence of the hindrance of free rotation due to the high order of the $(S_2C)$-$(NEt_2)$ bond[25,47], as observed for DMF[27]. On the other hand, in the spectrum of DEDTC only one signal is observed for both $CH_2$ and $CH_3$, indicating the equivalence of the protons. When DTS is broken, the two fragments are equivalent (DEDTC anion) and the resonant form III, which allows the free rotation of $(S_2C)$-$(NEt_2)$ bond, predominates. Therefore, in the spectra of $[Pd(S_2CNEt_2)_2]$ **(3)** and $[Pt(S_2CNEt_2)_2]$ **(5)** the quadruplets and triplets observed were assigned to $CH_2$ and to $CH_3$ respectively. Both groups are equivalent, as confirmed by the X-ray results. The upfield shifts observed for all the protons in comparison with those corresponding to DEDTC are also present in other DEDTC complexes[27]. In contrast, in the spectrum of $[PtCl_2(DTS)]_2$ **(4)**, the non-equivalence of the protons in $CH_2$ and $CH_3$ is evident, which confirms the presence of DTS in the complex. The resonances appear as upfield-shifted, broad multiple signals in comparison to those of DTS. The double bond character for C-N in the complex is greater than the corresponding to DTS[27] and as a consequence the hindrance to rotation increases. This observation is consistent with the IR results.

**Table 3.** Fractional atomic coordinates ($x10^4$) with the equivalent tempertaure factors.

| | X/A | Y/B | Z/C | Beq |
|---|---|---|---|---|
| $Pd_1$* | 10000 | 0 | 5000 | 3.44 |
| $Pd_2$* | 0 | 5000 | 10000 | 3.46 |
| $S_{11}$ | 8714(1) | 1171(1) | 4641(1) | 4.62 |
| $S_{12}$ | 9149(1) | -2825(1) | 5348(1) | 4.32 |
| $S_{21}$ | -347(1) | 2178(1) | 9148(1) | 4.30 |
| $S_{22}$ | 358(1) | 6171(1) | 8714(1) | 4.65 |
| $C_{11}$ | 8360(1) | -1274(4) | 5004(2) | 3.89 |
| $N_{12}$ | 7594(1) | -1836(4) | 4988(1) | 4.38 |
| $C_{13}$ | 7334(2) | -3914(6) | 5337(2) | 5.99 |
| $C_{14}$ | 6961(2) | -447(6) | 4635(2) | 5.70 |
| $C_{15}$ | 6890(2) | -791(8) | 3710(2) | 6.84 |
| $C_{16}$ | 7097(3) | -3635(9) | 6237(3) | 8.48 |
| $C_{21}$ | 7(2) | 3759(4) | 8355(1) | 3.86 |
| $N_{22}$ | 4(2) | 3167(4) | 7600(1) | 4.52 |
| $C_{23}$ | 363(2) | 4578(6) | 6966(2) | 5.58 |
| $C_{24}$ | -335(2) | 1112(6) | 7333(2) | 5.93 |
| $C_{25}$ | 1280(3) | 4214(8) | 6881(2) | 6.95 |
| $C_{26}$ | -1224(3) | 1322(9) | 7096(3) | 8.31 |
| $H_{131}$ | 6812(2) | -4499(6) | 5005(2) | 8.46 |
| $H_{132}$ | 7826(2) | -5054(6) | 5285(2) | 8.46 |
| $H_{141}$ | 6383(2) | -814(6) | 4915(2) | 8.46 |
| $H_{142}$ | 7115(2) | 1209(6) | 4750(2) | 8.46 |
| $H_{151}$ | 6348(2) | 38(8) | 3512(2) | 8.46 |
| $H_{152}$ | 6820(2) | -2490(8) | 3605(2) | 8.46 |
| $H_{153}$ | 7408(2) | -206(8) | 3372(2) | 8.46 |
| $H_{161}$ | 6806(3) | -5102(9) | 6429(3) | 8.46 |
| $H_{162}$ | 6662(3) | -2338(9) | 6255(3) | 8.46 |
| $H_{163}$ | 7598(3) | -3278(9) | 6641(3) | 8.46 |
| $H_{231}$ | 76(2) | 4218(6) | 6390(2) | 8.46 |
| $H_{232}$ | 255(2) | 6236(6) | 7121(2) | 8.46 |
| $H_{241}$ | 7(2) | 527(6) | 6817(2) | 8.46 |
| $H_{242}$ | -292(2) | -26(6) | 7827(2) | 8.46 |
| $H_{251}$ | 1457(3) | 4975(8) | 6317(2) | 8.46 |
| $H_{252}$ | 1374(3) | 2505(8) | 6833(2) | 8.46 |
| $H_{253}$ | 1645(3) | 4842(8) | 7373(2) | 8.46 |
| $H_{261}$ | -1509(3) | -176(9) | 6931(3) | 8.46 |
| $H_{262}$ | -1245(3) | 2409(9) | 6585(3) | 8.46 |
| $H_{263}$ | -1544(3) | 2021(9) | 7605(3) | 8.46 |

* atoms fixed at special positions

The most spectacular difference in the $^{13}C$ NMR spectra can be found in the chemical d corresponding to C=S. In tetraethylthiouram disulfide this signal appears at 192.67 ppm and the coordination to the platinum **(4)** produces a shift to upper fields. In the DEDTC the signal appears at 207.29 ppm but in the complexes **(3)** and **(5)** it shifts down-field. In the spectrum of $[PtCl_2(DTS)]_2$ **(4)**, all the signals are split due to the non-equivalence of the two halves of the DTS molecule[27] or to the presence of two structurally non-equivalent molecules of DTS. Both $CH_2$ and $CH_3$ carbon atoms in **(3)** and **(5)** are equivalent as expected for DEDTC complexes. In the platinum complex the shifts are slightly higher than in the palladium complex which is consistent with the higher acceptance ability of the platinum.

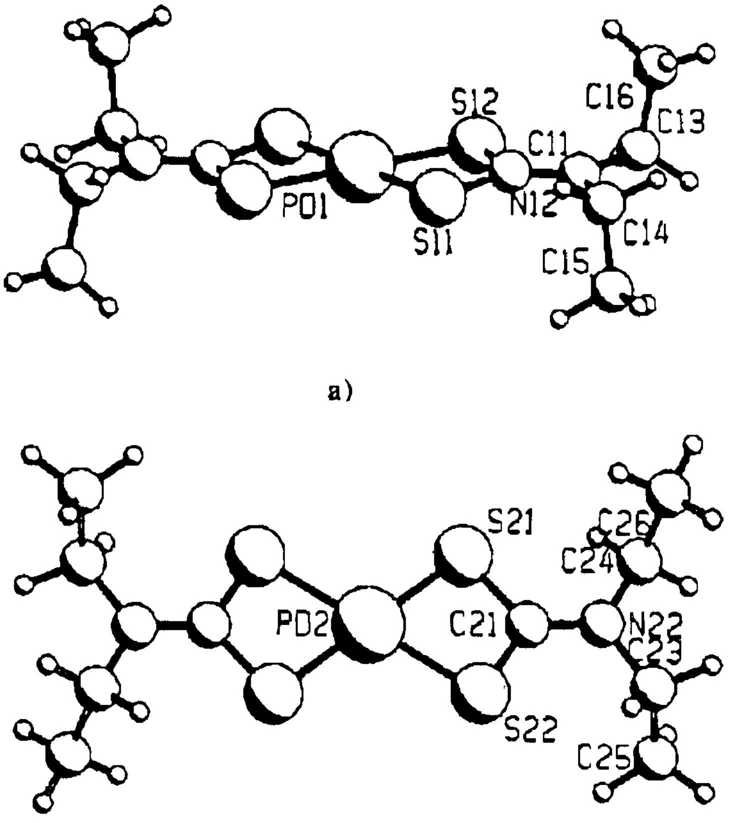

**Figure 1.** Two crystallographically non-equivalent molecules in the structure off $[Pd(S_2CNEt_2)_2]$. Figures a) and b) show the different orientation of the ethyl groups.

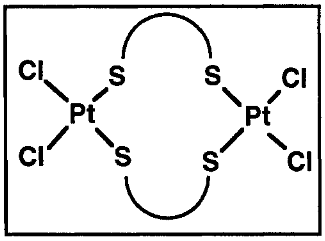

**$(PtCl_2DTS)_2$**

(4)

**Scheme 5. Structure proposed for the dimer Pt(II)-tetraethyldithiouram disulfide complex**

### *$^{1}H$, $^{13}C$ NMR and $^{195}Pt$ Spectra*

The $^{1}H$ and $^{13}C$ NMR spectra of tetraethylthiouram disulfide, the sodium diethyldithiocarbamate and the Pd(II) and Pt(II) complexes **(3)**, **(4)** and **(5)** are given in Table 5.

The $^{195}Pt$ NMR spectrum of $[PtCl_2(DTS)]_2$ gave only one signal at -1763 ppm ($K_2PtCl_6$ as reference).This resonance appears slightly shifted as expected for a $PtCl_2S_2$ environment, which could be attributed to the anomalous charge density arrangement on the sulfur atom in DTS before described. The appearance of only one signal indicates that the arrangement of the two platinum atoms in the dimer is equivalent and that only one specie is present in solution[48].

**Table 4.** Bond Lengths (Å) and Bond Angles (°) with their e.s.d.'s for $[Pd(S_2CNEt_2)_2]$

| | | | |
|---|---|---|---|
| $S_{11}$-$Pd_1$ | 2.312 (1) | $S_{12}$-$Pd_1$-$S_{11}$ | 75.6 (0.1) |
| $S_{12}$-$Pd_1$ | 2.321 (1) | $S_{22}$-$Pd_2$-$S_{21}$ | 75.6 (0.1) |
| $S_{21}$-$Pd_2$ | 2.320 (1) | $C_{11}$-$S_{11}$-$Pd_1$ | 86.6 (0.1) |
| $S_{22}$-$Pd_2$ | 2.311 (1) | $C_{11}$-$S_{12}$-$Pd_1$ | 86.9 (0.1) |
| $C_{11}$-$S_{11}$ | 1.738 (3) | $C_{21}$-$S_{21}$-$Pd_2$ | 86.5 (0.1) |
| $C_{11}$-$S_{12}$ | 1.713 (2) | $C_{21}$-$S_{22}$-$Pd_2$ | 87.3 (0.1) |
| $C_{21}$-$S_{21}$ | 1.735 (2) | $S_{12}$-$C_{11}$-$S_{11}$ | 110.8 (0.1) |
| $C_{21}$-$S_{22}$ | 1.715 (2) | $N_{12}$-$C_{11}$-$S_{11}$ | 123.4 (0.2) |
| $N_{12}$-$C_{11}$ | 1.307 (3) | $N_{12}$-$C_{11}$-$S_{12}$ | 125.7 (0.2) |
| $C_{13}$-$N_{12}$ | 1.479 (4) | $C_{13}$-$N_{12}$-$C_{11}$ | 120.3 (0.2) |
| $C_{14}$-$N_{12}$ | 1.472 (4) | $C_{14}$-$N_{12}$-$C_{11}$ | 122.0 (0.2) |
| $C_{16}$-$C_{13}$ | 1.540 (6) | $C_{14}$-$N_{12}$-$C_{13}$ | 117.7 (0.2) |
| $C_{15}$-$C_{14}$ | 1.539 (5) | $C_{16}$-$C_{13}$-$N_{12}$ | 110.2 (0.3) |
| $N_{22}$-$C_{21}$ | 1.294 (3) | $C_{15}$-$C_{14}$-$N_{12}$ | 111.2 (0.2) |
| $C_{23}$-$N_{22}$ | 1.486 (4) | $S_{22}$-$C_{21}$-$S_{21}$ | 110.6 (0.1) |
| $C_{24}$-$N_{22}$ | 1.465 (4) | $N_{22}$-$C_{21}$-$S_{21}$ | 123.8 (0.2) |
| $C_{25}$-$C_{23}$ | 1.530 (6) | $N_{22}$-$C_{21}$-$S_{22}$ | 125.6 (0.2) |
| $C_{26}$-$C_{24}$ | 1.517 (6) | $C_{23}$-$N_{22}$-$C_{21}$ | 120.1 (0.2) |
| | | $C_{24}$-$N_{22}$-$C_{21}$ | 122.6 (0.2) |
| | | $C_{24}$-$N_{22}$-$C_{23}$ | 117.3 (0.2) |
| | | $C_{25}$-$C_{23}$-$N_{22}$ | 111.6 (0.3) |
| | | $C_{26}$-$C_{24}$-$N_{22}$ | 111.5 (0.3) |

**Table 5**· $^{1}H$ and $^{13}C$ NMR shifts (ppm) of Pd(II).and Pt(II) DEDTC and DTS complexes*.

**$^{1}H$ NMR**

| Compound | $CH_2$ | $CH_3$ |
|---|---|---|
| **DTS** | 4.03 m | 1.50 t<br>1.32 t |
| **$[PtCl_2(DTS)]_2$** | 3.65 m | 1.36 m |
| **Na-DEDTC** | 4.02 q | 1.23 t |
| **$[Pd(S_2CNEt_2)_2]$** | 3.73 q | 1.29 t |
| **$[Pt(S_2CNEt_2)_2]$** | 3.57 q | 1.29 t |

**$^{13}C$ NMR**

| Compound | C=S | $CH_2$ | $CH_3$ |
|---|---|---|---|
| **DTS** | 192.67 | 52.02<br>47.60 | 13.26<br>11.46 |
| **$[PtCl_2(DTS)]_2$** | 186.03<br>185.71 | 45.24<br>44.64 | 12.45<br>12.39 |
| **Na-DEDTC** | 207.29 | 48.00 | 12.71 |
| **$[Pd(S_2CNEt_2)_2]$** | 210.05 | 44.03 | 12.42 |
| **$[Pt(S_2CNEt_2)_2]$** | 215.07<br>208.05 | 44.46 | 12.55 |

*** in $CDCl_3$**

***Acknowledgements***

*We are grateful to DGICYT Ref. PB94-0922-C02-01, to European Community HCM, ref. ERBCHRXCT 920016 and to Johnson Matthey for $K_2PdCl_4$ and $K_2PtCl_4$ supplied.*

### References

1. N. Farrell, *Transition Metal Complexes as Drugs and Chemotherapeutic Agents,* Kluwer Academic Publishers, Dordrecht, 1989.
2. T.F.Slater, M. Ahmed, and S.A. Ibrahim, *J.Clin.Hematol.Oncol.*, **7**, 534 (1977).
3. C.L. Litterst, S. Tong, Y. Hirokata, and Z.H. Siddik, *Cancer Chemother. Pharmacol*, **8**, 67 (1982).
4. B. Odenheimer, and W. Wolf, *Inorg. Chim. Acta.*, **66**, L41 (1982).
5. B. Leyland-Jones, C. Morrow, S. Tate, C. Urmacher, C. Gordon, and C.W. Young, *Cancer Research,* **43**, 6072 (1983).
6. C.L. Litterst, F. Bertolero, and J. Uozumi, *Assoc. Int. Cancer Res. Symp.*, **4**. 227 (1985).
7. J.E. Melvik, and E. O. Pettersen, *Inorg. Chim. Acta,* **137**, 115 (1987).
8. P. C. Dedon, and R. F. Borch, *Biochem. Pharmacol.*, **36**, 1955 (1087).
9. B.J. Corden, *Inorg. Chim. Acta*, **137**, 125 (1987).
10. T.G. Appleton, J.W. Connor, J.R. Hall, and P.D. Prenzler, *Inorg. Chem.,* **28**, 2030 (1989).
11. A. Caubet, V. Moreno, E. Molins, and C. Miravitlles, *J. Inorg. Biochem.*, **48**, 135 (1992).
12. P.S. Murdoch, J.D. Ranford, P.J. Sadler, and S.J. Berners-Price, *Inorg. Chem*, **32**, 2249 (1993).
13. O.M. Ni Dhubhghaill, P.J. Sadler, and E. Garcia Fernandez, *Metal Based Drugs*, **2**, 19 (1995).
14. R.F. Borch, D.L. Bodenner, and J.C. Katz, *Platinum Coordination Complexes in Cancer Chemotherapy* (eds. M.P. Hacker, E.B. Douple, and I.H. Krakoff) p. 154, Martinus Nijhoff, Boston (1984)
15. P.K. Gessner and T. Gessner, *Disulfiram and its metabolite, Diethyldithiocarbamate. Pharmacology and status in the treatment of alcoholism, HIV infections, AIDS and heavy metal toxicity.* Chapman & Hall, London, 1992.
16. G. Celichowski, L. Marlielewski and S. Plaza, *Analyst,* **120**, 2273 (1995)
17. R.E. Norman and P. Sadler, *Inorg.Chem.*, **27**, 3583 (1988).
18. R.F. Borch, J.C. Katz, P.H. Lieder, and M.E. Pleasants, *Proc. Natl. Acad. Sci. USA,* **77**, 5441 (1980).
19. E.L. Lempers and J. Reedijk, *Inorg. Chem.*, **29**, 217 (1990).
20. T.M. Kitson, *Biochem. J.* **278**, 189 (1991).
21. P.A. Andrews, W.E. Wung, and S.B. Howell, *Analytical Biochemistry,* **143**, 46 (1984).
22. P.J. Nichols and M.W. Grant, *Aust. J. Chem.*, **35**, 2455 (1982).
23. A. Furlani, V. Scarcia, G. Faraglia, L. Sindellari, L. Trincia and M. Nicolini, *Eur. J. Med. Chem.*, **21**, 261 (1986).
24. I.L. Karle, J.A. Estlin and K. Britts, *Acta Cryst.* **22**, 273 (1967).
25. Y. Wang, and J.H. Liao, *Acta Cryst.* **B45**, 65 (1989).
26. P.J. Nichols and M.W. Grant, *Aust. J. Chem.*, **36**, 1379 (1986).
27. H. Brinkhoff, A. Grotens and J. Steggerda, *Rec.Trav. Chim. Pays-Bas.*, **89**, 11 (1970).
28. I. Cuadrado and M. Moran, *Trans. Met. Chem.*, **11**, 375 (1986).
29. G. Contreras and H. Cortes, *J. Inorg. Nucl. Chem.*, **33**, 1337 (1971).
30. A.V. Babaeva, G.V. Derbisher and G.G. Afanaseva, *Russ. J. Inorg. Chem.*, **7**, 6 (1962); A.V. Babaeva and G.V. Derbisher, *Russ. J. Inorg. Chem.*, **7**, 1401 (1962).
31. B. Skelton and A. White, *Aust. J. Chem.*, **30**, 1693 (1977).
32. M. Bonamico, G. Dessy, C. Mariani, A. Vaciago, and L. Zambonelli, *Acta Cryst.* **19,** 619 (1965).
33. M. Bonamico, G. Dessy, A. Mugnoli, A. Vaciago, and L. Zambonelli, *Acta Cryst.* **19,** 886 (1965).
34. M. Bonamico, G. Mazzone, A. Vaciago, and L. Zambonelli, *Acta Cryst.* **19,** 898 (1965).
35. A.Z. Amanov, G.A. Akukina and M.A. Porai-Koshits, *J. Struct. Chem.*, **8,** 149 (1967).
36. T.O. Kocaba, C.G. Young, and E.R.T. Tiekink, *Inorg. Chimica Acta,* **174,** 143 (1990).
37. P. Main, S.J. Fiske, S.E. Hull, L. Lessinger, G. Germain, J.P. Declercq, and M.M. Woolfson, *MULTAN* 11/82, York University, England and Louvain, Belgium, 1982.
38. G.M. Sheldrick, *SHELX*-76 Cambridge University, England, 1976.
39. L. Marcheselli, C. Preti, M. Tagliazucci, V. Cherchi, L. Sindellari, A. Furlani, A. Papaioannou and V. Scarcia, *Eur. J. Med.Chem.*, **28**, 347 (1993).
40. G. Contreras, G. Seguel and J. Alderete, *Spectrochim. Acta,* **50A**, 371 (1974).
41. K. Jensen, B. Dahl, P. Nielsenl and G. Borch, *Acta Chem.Scand.*, **25**, 2029 (1971).
42. F. Forghieri, C. Preti, L. Tassi and G. Tosi, *Polyhedron*, **7**, 1231 (1988).
43. F. Bonati and R. Ugo, *J. Organometal. Chem.*, **10**, 257 (1967).
44. J. Criado, J. Salas, M. Medarde, I. Fernández and B. Macías, *Inorg. Chim. Acta,* **174,** 67 (1990).
45. L. Sindellari, G. Faraglia, L. Trincia, A. Furlani and V. Scarcia, *Inorg.Chim.Acta,* **106**, 31 (1985).
46. A.T. Baker and M.T. Emmet, *Aust.J . Chem.*, **45**, 42 (1992).
47. J. Richards, D. Tarbell and E. Hoffmeister, *Tetrahedron*, **24**, 6485 (1968).
48. T.G. Appleton, J.R. Hall and D.W. Neale, *Inorg.Chim.Acta*, **104**, 19 (1985).

**Received: August 11, 1997 - Accepted: August 19, 1997 - Received in revised camera-ready format: October 30, 1997**

